# Overview of Botanical Status in EU, USA, and Thailand

**DOI:** 10.1155/2013/480128

**Published:** 2013-10-21

**Authors:** Weena Jiratchariyakul, Gail B. Mahady

**Affiliations:** ^1^Department of Pharmacognosy, Faculty of Pharmacy, Mahidol University, Sri Ayutthaya Road, Rajthevi, Bangkok 10400, Thailand; ^2^Clinical Pharmacognosy Laboratory, College of Pharmacy, PAHO/WHO Collaborating Centre for Traditional Medicine, University of Illinois, Chicago, IL, USA

## Abstract

The botanical status in EU, USA, and Thailand is different owing to the regulatory status, the progress of science, and the influence of culture and society. In the EU, botanicals are positioned as herbal medicinal products and food supplements, in the US they are regulated as dietary supplements but often used as traditional medicines, and in Thailand, they are regulated and used as traditional medicines. Information for some of the most popular botanicals from each country is included in this review.

## 1. Introduction

Currently botanicals, herbal medicines, herbal medicinal products, botanical dietary supplements, and traditional medicines are diversely defined according to the regulations of the country in which the product is manufactured and its final destination. In the European Union (EU) and USA, the extracts and extract fractions (special extracts) are primarily used as active compositions in the products, which are regulated as herbal medicinal products (HMPs), and food supplements in EU, and dietary supplements in USA. In Asia, the crude drugs are often used as active composition in the products, which are usually classified as traditional medicines. Herbal medicinal products are intended for use as modern and traditional drugs or as food supplements that are used to maintain heath. In Thailand, as a representative of Asian countries, the botanical products are regulated both as traditional medicines and health foods. Complementary and alternative medicine also includes the use of botanicals in prevention and therapy of disease states. The progress in science and technology of botanicals started approximately between 1811 and 1815, when the term “pharmacognosy” was firstly used in EU [1]. The purpose of this review is to give a brief overview of the regulatory status of botanicals and some of the more recent data on the therapeutic effects of some of the most popular botanicals in these countries.

## 2. Botanicals in EU

The advancement of botanicals in Germany resulted from the improvement of the drug act in 1976, which required the evidence for the registration to support the quality, effectiveness, and safety of the drugs, including herbal medicines. Herbal medicines are classified as special drugs because of the difficulties in providing evidence of quality and effectiveness. In the later 1970s, the German Ministry of Public Health appointed the Commission E, a group that was comprised of experts in herbal drugs and preparations from medicinal plants. In 1978, the Commission E reviewed botanicals and issued the evaluation criteria for the safety and efficacy of botanicals as follows [2].

1. The traditional use and the long use of botanicals may indicate the safety and the potential effectiveness of the botanicals.
2. The chemical evidence, especially the specific active substance, may indicate the potential activity and/or the toxicity of the active substance. However, it depends on the type and the quantity of the active substance.
3. *In vitro* and *in vivo* pharmacology and toxicity studies of the botanical extracts and the isolated active substances provide evidence for the potential use of the botanicals. This result is very useful where there are no results from clinical studies.
4. The resulting data from the clinical studies confirms the safety and the efficacy of botanicals.
5. The field and epidemiological studies provide relevant information about the duration of the species particular environment, which are useful for the evaluation of botanical safety and effectiveness.
6. The case reports from the physicians are also useful for the evaluation of the botanical effectiveness. However, the evidence is not as strong as the controlled clinical studies.
7. The unpublished information and study from the manufacturers are also useful for the evaluation.

The Commission E issued botanical monographs until the year 1995, when the European Union started to harmonize drug regulations. At that point, the Commission E monographs became historical documentation and a basis for the scientific documentation of botanicals [3].

One of the most popular botanicals in EU is *Ginkgo biloba* L. [4–6]. It is thought that, in the EU, Ginkgo became extinct during the ice era but was reintroduced to EU in the eighteenth century from East Asia, where the Ginkgo leaf had been traditionally used as a remedy for bronchial asthma and topically as a wound-plaster.

*Ginkgo biloba*, the last plant in the family Ginkgoaceae, is thought to be one of the oldest trees still surviving on earth [4–6]. Some specimens are over 30–40 meters high and several hundred years of age. The leaf form looks like two lobes of a spreading fan or maiden's hair. *Ginkgo biloba *has many common names, one of which is the maidenhair tree. The body of research data for *Ginkgo* is extensive, and research on various aspects of the plant safety, quality, and efficacy is currently ongoing. Nowadays, the plant is cultivated in Japan, South Korea, USA (South California), and France (Bordeaux).

Monographs for Ginkgo leaf (Folium Ginkgo; Ginkgo Folium) are present in many pharmacopoeias and official publications worldwide including the Chinese Pharmacopoeia, the European Pharmacopoeia, the USA Pharmacopoeia, and the WHO monographs. In terms of chemistry, there are several groups of compounds present in Ginkgo leaves. They include the following.

**Figure 1:**
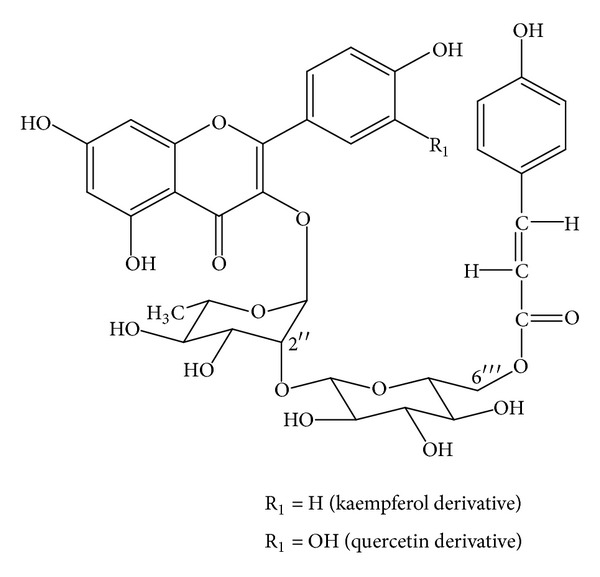
Two major flavonol-acyl-glucosides of *Ginkgo biloba* [7].

**Figure 2:**
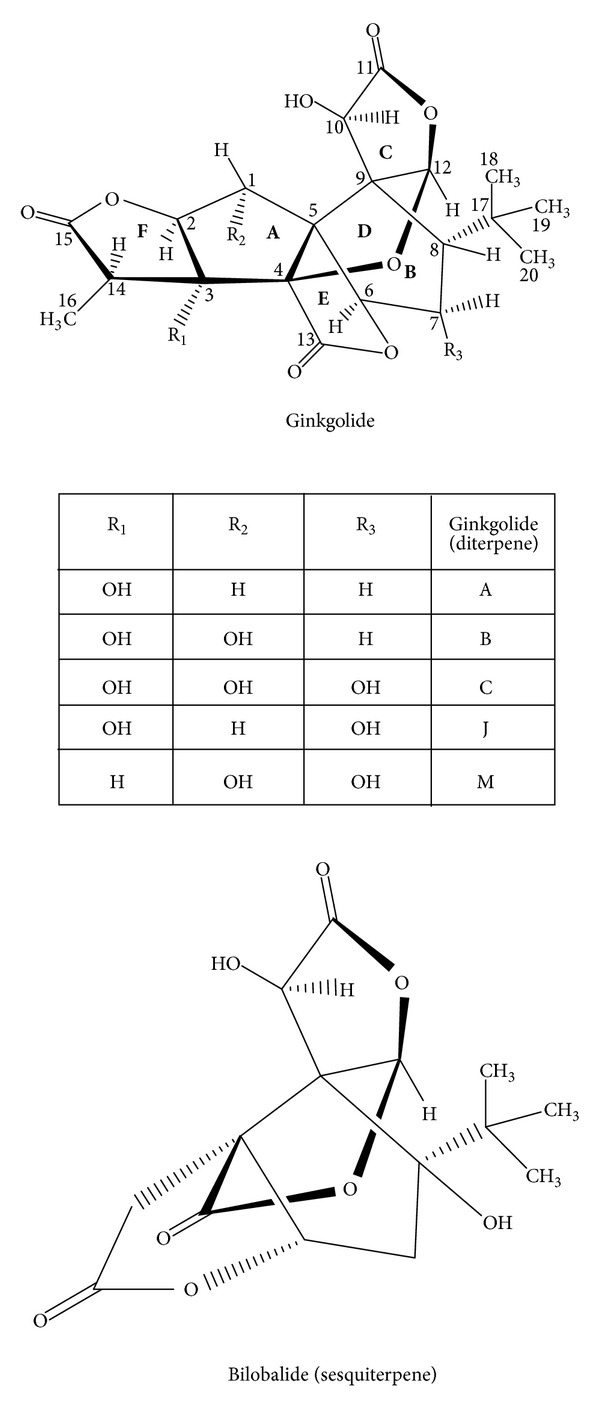
Terpene lactones, ginkgolides and bilobalide, of *Ginkgo biloba* [7].

1. Flavonoid glycosides: mono-, di-, and triglycosides (0.5–1.8%), including quercetin- and isorhamnetin-3-*O*-glucosides, kaempferol-7-*O*-glucoside, quercetin-3-*O*-rhamnoside, 4′-*O*-methyl-myricetin-3-*O*-glucoside, kaempferol-3-*O*-rutinoside, kaempferol-3-*O*-glucorhamnoside, quercetin-3-*O*-rutinoside, 4′-*O*-methyl-myricetin-3-*O*-rutinoside, syringetin-3-*O*-rutinoside, kaempferol-3-*O*-glucorhamnoside, and quercetin-3-*O*-glucorhamnoside.
2. Flavonol-acyl-glucosides (0.06–0.2%): kaempferol- and quercetin-3-*O*-[6-p-coumaroyl-glucorhamnoside] (Figure 1).
3.   Biflavonoids (0.4–1.9%): amentoflavone, bilobetin, 5-methoxy-bilobetin, ginkgetin, isoginkgetin and sciadopitysin.
4. Catechins (up to 0.04%) and oligomeric proanthocyanidins (8–12%), of which the major skeletons belong to delphinidin and cyanidin. In addition (+)-catechin, (−)-epicatechin, (+)-gallocatechin, and (−)-epigallocatechin are also found.
5. Terpenoids include diterpenes (ginkgolides A, B, C, J, and M; up to 0.23%) and sesquiterpenes (bilobalide; up to 0.2%) (Figure 2).
6. Ginkgo terpenoids containing three groups of lactones and a tertiary butyl group cause the bitterness of the ginkgo extract.
7. Steroids include sitosterol, campesterol, 22-dihydrobrassicasterol, and sitosterylglucoside.
8. Organic acids include shikimic acid, 3-methoxy-4-hydroxy benzoic acid, 4-hydroxy benzoic acid, and ginkgolic acid.
9. Nitrogen containing compounds.

The first nitrogen containing compound, 6-hydroxy-kynurenic acid, was recently found in Ginkgo leaf. The quantity of this compound depends on the plant age. The leaves collected in the fall contain the maximum content (up to 0.24%) of this compound, which is the major metabolite of tryptophan.

The active composition or drug material from *G. biloba *leaf is prepared using 60% aqueous acetone as extraction solvent; the biflavonoids and ginkgolic acid are removed, and the drug-extract ratio is 50 : 1 (average). The extract fraction or special extract of *G. biloba* leaf contains ginkgo flavones glycosides (22–27%) and terpene lactones (ginkgolides and bilobalide) (5–7%). The level of ginkgolic acid must not exceed 5 ppm.

The special extract of Ginkgo leaf possesses the following pharmacological activities: increases the tissue resistance to hypoxia, especially the brain tissue to the oxygen-deficient blood; it inhibits the brain edema; decreases retinal edema and lesions; it inhibits aging and the reduction of muscarinic cholinergic receptor and *α*_2_-adrenergic receptor and it increases choline uptake in the hippocampus; it increases cognition by enhancing memory and learning; it increases blood circulation, especially microcirculation; it improves the blood flow; it removes toxic oxygen radicals (by flavonoids); it inhibits the platelet activating factor, PAF (by ginkgolides), and it provides neuroprotection (by ginkgolides A and B; bilobalide).

The special extract from Ginkgo leaf has several mechanisms of action leading to various therapeutic uses. An important use of the extract is indicated for age associated memory impairment, also known as cerebral insufficiency. The symptoms cover the following: lack of concentration, memory loss, sensitivity, being easily tired, lack of interest, being depressed, being anxious, being dizzy, tinnitus, and headache. Some symptoms indicate that a disorder of cerebral blood flow is the beginning of the oxygen-deficiency in brain and degenerative dementia.

Observed adverse events of Ginkgo leaf are rare. They include headache, dizziness, palpitations, gastrointestinal disorders, and skin allergies. Ginkgolic acid is a potent contact allergen; therefore, the ginkgolic acid level in the extract must not exceed 5 ppm. The special Ginkgo extract is not mutagenic or carcinogenic and is nontoxic to the reproductive system.

The patients can drive or work with machinery when they take Ginkgo extracts. The injectable form of Ginkgo extract is not allowed due to potential allergic reactions, which may include serious symptoms such as arrhythmia and anaphylaxis.

The therapeutic indications allowed for Ginkgo leaf extracts by the German Ministry of Health are as follows.

1. Relief of the symptoms and uneasy feeling from the cerebral insufficiency and degenerative dementia: rhe drug administration: 120–240 mg, 2-3 times a day, for at least 8 weeks. After 3 months the physician should consider the continuation of the drug administration.
2. For patients with peripheral arterial occlusive diseases, along with walking therapy: the drug administration: 120 mg, 3 times a day, for at least 6 weeks.
3. Tinnitus, dizziness: the drug administration: 160 mg, 3-4 times a day; the administration longer than 6–8 weeks is not necessary.

### 2.1. Botanicals as Food Supplement and Special Food [7, 8]

 Besides carbohydrates, fats, and proteins, humans also need secondary metabolites from botanicals. These botanicals make food colorful (carrot, tomato, etc.) and digestible (spices). The citrus extract with bioflavonoids, the extract of *Allium ursinum*, and the extract of *Linum usitatissimum *are the examples of food supplements. Some botanicals are used as special foods in the chronic diseases, such as cardiovascular, hypercholesterolemia, diabetes, rheumatoid arthritis, and osteoporosis. Red wine and its protective phenols are used for preventing the cardiovascular diseases. Soy and its estrogenic isoflavones are used as alternative for steroidal hormones in the climacteric period.

## 3. Botanical Dietary Supplements in the USA

For the past thirty years, the consumption of botanical dietary supplements (herbal preparations) by USA consumers has increased, and approximately half of adults in the USA use at least one dietary supplement [9]. Supplement sales in the USA were estimated at $11.5 B in 2012, an increase of seven percent [10]. The most common reasons cited for using dietary supplements are to improve overall health as well as maintain health [9]. Other reasons for increased use of dietary supplements included improvements in bone health for women, mental health, prostate health for men, weight loss, and menopause or hot flashes for women [9]. Many botanical dietary supplements (BDS) are also used by the USA population for the treatment or prevention OF a wide array of ailments including the common cold, depression, and other nonlife-threatening medical conditions, although they are not regulated for this purpose [9, 10]. Consumers over the age of 65, baby boomers, and adolescents make up the major of consumers using dietary supplements. Interestingly, condition-specific products are increasingly being used, perhaps due to the older age populations buying these products. Some of the categories of dietary supplements that are very popular in the USA include the digestive supplements (probiotics), omega-3 fatty acids, joint support, and eye health, all which increased in market share in 2012 [9]. 

The increasing use of condition specific products is interesting in that dietary supplements are regulated according to the Dietary Supplements Health and Education Act of 1994, which states that dietary supplements are not to be used for treating any condition or disease state but only as support for maintaining and promoting good health [11]. Each dietary supplement package must state that the product is a “Dietary Supplement,” and *“These statements have not been evaluated by the Food and Drug Administration. This product is not intended to diagnose, treat, cure, or prevent any disease.”* Thus, it appears that regardless of how supplements are regulated in the USA, consumers still use these products to manage symptoms and treat specific conditions. This is problematic as many dietary supplements do not have sufficient data to support efficacy, and in some cases there are contraindications, drug interactions, and adverse effects associated with the use of specific supplements, of which consumers are not fully aware. 

According to the National Center for Complementary and Alternative Medicine at the National Institutes of Health, the five most searched-for botanical dietary supplements of 2012 included Aloe vera, Echinacea, evening primrose oil, fenugreek, and St. John's wort [11]. We have included a brief overview of each of these botanicals; for a more detailed review, including pharmacopoeial details, reviews of pharmacology, and chemistry, please refer to the WHO monographs on selected medicinal plants, volumes 1–4 [12–15] (WHO 2009). 

### 3.1. Aloe Vera: *Aloe vera* (L.) Burm. f. (Liliaceae)

**Figure 3:**
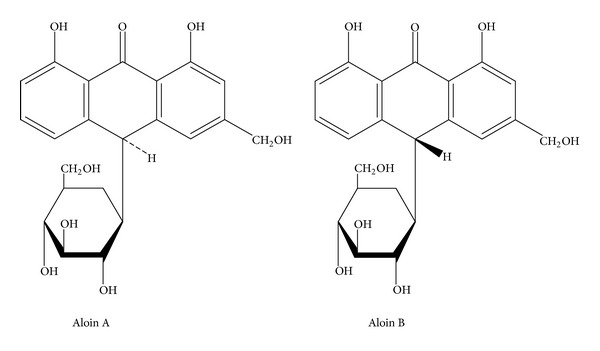
The structures of aloin A and B, two of the major constituents of *Aloe vera* latex.

*Aloe vera* is a succulent perennial that resembles a cactus and is recognized by many common names including aloe, Aloe Vera, aloes, cape aloe, and curacao aloe [12]. This plant is the source of two botanically distinct drugs, “aloe” which is a bitter yellow latex originating in the cells of the pericycle and adjacent leaf parenchyma, and *Aloe vera* gel, which is a colorless mucilage obtained from the parenchymatous cells in the leaves. The biologically active chemical constituents of the yellow latex are the hydroxyanthracene derivatives, aloin A and aloin B (Figure 3), whereas the gel consists primarily of water and polysaccharides (pectins, hemicellulose, glucomannan, acemannan, and mannose derivatives) [12, 16, 17].

Clinically, aloe (solidified yellow latex) is used for the short-term treatment of occasional constipation [12]. It should not be used continuously for longer than 1-2 weeks and may cause adverse reactions, including abdominal spasms and pain which can occur even after a single dose [12, 16]. Overdose can lead to abdominal spasms and pain, as well as the formation of watery stool. Chronic abuse of this drug as a laxative can lead to hepatitis, electrolyte disturbances (hypokalaemia and hypocalcaemia), metabolic acidosis, malabsorption, weight loss, albuminuria, haematuria, weakness, and orthostatic hypotension [12, 16]. Another adverse effect is secondary aldosteronism that may also occur after chronic use. Aloe, as a laxative, is contraindicated in patients with intestinal obstruction or stenosis, atony, severe dehydration with electrolyte depletion, or chronic constipation. It should not be administered to patients with inflammatory intestinal disease, children under 10 years of age, during pregnancy, or during lactation (unless under the supervision of a physician) [12]. The safety of aloe latex was reported in 2007 [17]. This study showed that the administration of the latex to mice at a dose of 100 mg/kg for three months caused inflammation, genotoxicity, general toxicity, and sperm damage [17]. 

*Aloe vera* gel is also used in complementary and alternative medicine (CAM) to treat wounds and burns. In clinical trials, fresh *Aloe vera* gel has been shown to promote healing of burns, including radiation burns [12]. Clinical studies further suggest that fresh *Aloe vera* gel may promote wound healing; however, it should be noted that only fresh gel or preparations containing 10–70% fresh gel are active [18]. Dried or dated preparations are ineffective in either burn or wound healing. A 2012 systematic review that assessed the clinical trials using Aloe Vera gel for acute and chronic wounds suggested that the clinical trial data are in general of poor quality, biased, and conflicted [18]. *Aloe vera* gel has few adverse reactions and is only contraindicated in cases of known allergy to the Liliaceae [12, 16]. In terms of safety, recent studies in rodents have shown that *Aloe vera* gel is not genotoxic and has no toxic effects after 13 weeks of feeding [17, 19].

### 3.2. Echinacea: *Echinacea angustifolia* (DC.) Hell., *Echinacea pallida* (Nutt.) Nutt., and *Echinacea purpurea* (L.) Moench (Asteraceae)

**Figure 4:**
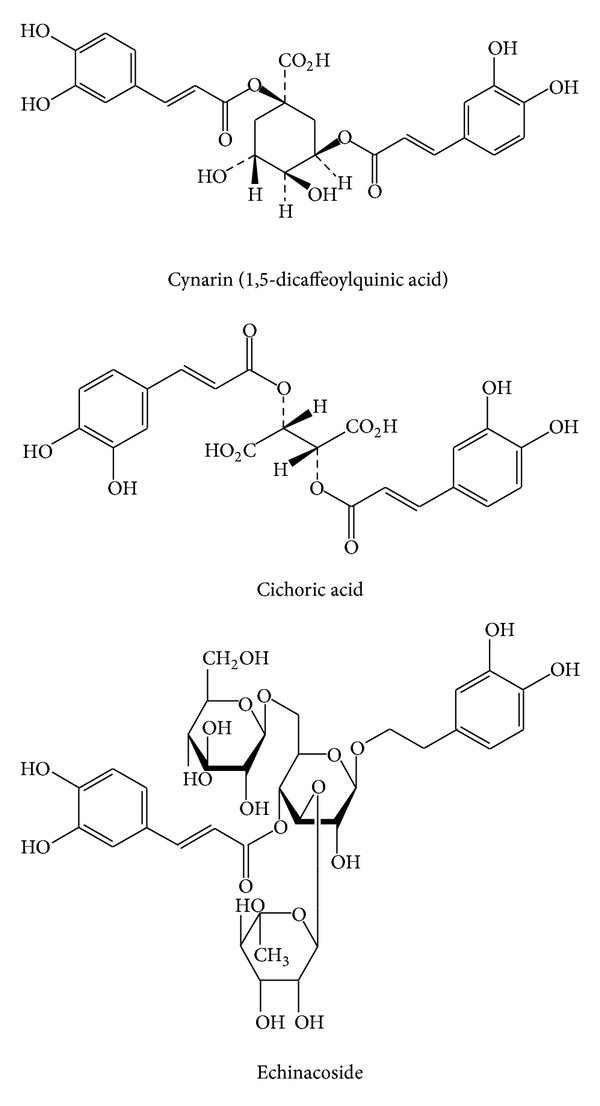
Structures of the caffeic acid derivatives that make up some of the major chemical compounds in *Echinacea*.

*Echinacea*, a native American plant and traditional medicine, is popular in the USA and Europe for the prevention and treatment of the symptoms of cold, flu, and upper respiratory infections [12, 16]. Clinical research suggests that *Echinacea* may enhance the immune response, but this effect is likely product specific, as certain preparations of *Echinacea*, including fresh pressed juice or isolated polysaccharides, appear to be more effective than other products [20, 21]. The chemistry of *Echinacea* is complicated but is well documented, and several groups of constituents, including alkamides and caffeic acid derivatives such as cichoric acid, echinacoside, cynarin, and cichoric acid methyl ester, are considered important for activity (Figure 4) [12].

A 2007 meta-analysis of 14 clinical trials evaluated the safety and efficacy of *Echinacea* on the duration and severity of the common cold [22]. The results of this analysis suggested that pretreatment with *Echinacea* reduced the odds of developing a cold by 58 percent and reduced the average duration of the infection by 1.4 days [22]. In the most recent clinical trials, the results assessing the effects of various *Echinacea* products on the severity and duration of the common cold are conflicted [23, 24]. The first clinical trial, using an unknown product for the treatment and prevention of the common cold, resulted in a negative outcome [23], where administration of *Echinacea* had no significant effect on the symptoms or duration of the common cold. The second study was a positive prevention trial [24] that showed a positive preventative effect of a specific product “Echinaforce” (Bioforce, Switzerland) on the common cold [24].

Interestingly, many of the clinical trials involving pediatric patients have negative outcomes as well as a higher incidence of skin rashes reported after treatment as an adverse effect [25]. Thus, at this time, it does not appear that there is any benefit from administering *Echinacea* products to children. In addition, people with allergies to the Asteraceae (ragweed and chamomile) may also have cross-sensitivity to *Echinacea* [12]. Furthermore, *Echinacea* products are not recommended during pregnancy. 

### 3.3. Evening Primrose Oil: *Oenothera biennis* L. (Onagraceae)

**Figure 5:**
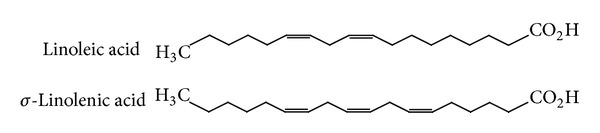
Structures of the fatty acid derivatives that make up some of the major chemical compounds in evening primrose oil.

Evening primrose oil (EPO) is the fixed oil extracted from the seeds of *O. biennis*, a native American wild flower introduced into Europe in the early 17th century [13]. EPO has been used internally for the management of atopic eczema, diabetic neuropathy, premenstrual syndrome, and cyclic mastalgia [13]. Mastalgia is a common cyclic breast condition, with swelling and breast pain being severe enough to interfere with usual daily activities. The major chemical constituents of the oil include *cis*-linoleic acid (65–80%), *γ*-linoleic acid (8–14%) (Figure 5), oleic acid (6–11%), palmitic acid (7–10%), and stearic acid [13], most of which have antiinflammatory effects.

A review of the clinical trials for evening primrose oil up to the year 2001 is available in the WHO Monographs of Selected Medicinal Plants [13]. A more recent randomized, double-blind, placebo-controlled clinical trial involving 85 women with cyclic mastalgia showed that daily administration of EPO at a dose of 3000 mg per day (in divided doses) for 6 months decreased the pain and severity associated with this condition [26]. In terms of safety, EPO is known to inhibit platelet aggregation in animals; thus, patients taking anticoagulants should be closely monitored and checked by their physician prior to using products containing EPO. In terms of adverse effects, headaches, nausea, and minor diarrhea have all been reported [13]. In addition, EPO should be used with caution in patients with a history of epilepsy, as it may precipitate symptoms of undiagnosed temporal lobe epilepsy in schizophrenia patients taking epileptogenic drugs, particularly the phenothiazines [13]. 

### 3.4. Fenugreek: *Trigonella foenum-graecum* L. (Fabaceae)

**Figure 6:**
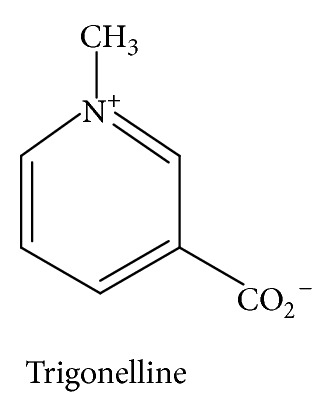
The structure of trigonelline, one of the chemical constituents of fenugreek.

*Trigonella foenum-graecum* L., commonly known as fenugreek, is a plant that has been extensively used as a source of antidiabetic compounds from its seeds and leaf extracts, including trigonelline (Figure 6) [27]. Preliminary human trials and animal experiments suggest possible hypoglycaemic and antihyperlipidemic properties of fenugreek seed powder when taken orally. One study showed that the action of fenugreek in lowering blood glucose levels is almost comparable to the effect of insulin [27]. In animal models, the soluble fiber of fenugreek appears to seed improve glucose homeostasis by delaying carbohydrate digestion and absorption and enhancing insulin action [28, 29]. 

However, the clinical data for fenugreek are currently inadequate to recommend it's use at this time and new clinical data for fenugreek soluble fiber are needed before any clinical recommendations can be made about its therapeutic use for diabetes [26]. 

### 3.5. St. John's Wort: *Hypericum perforatum* L. (Clusiaceae)

**Figure 7:**
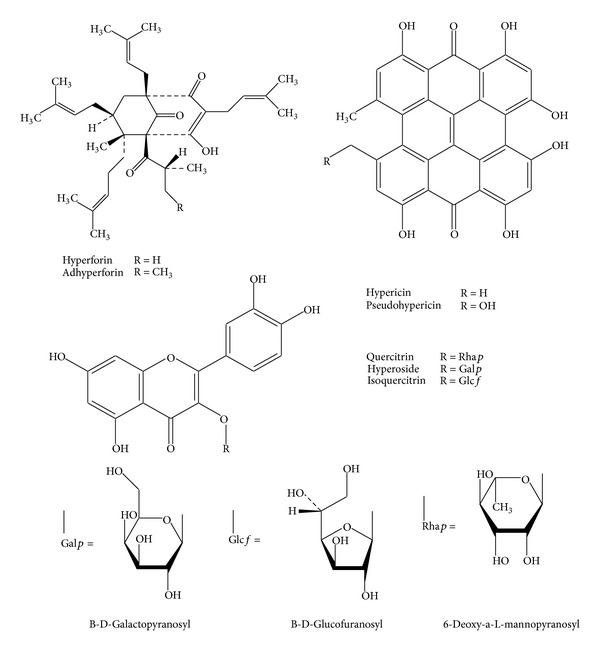
Structures of some of the major chemical constituents present in St. John's wort extracts.

*Hypericum perforatum *L. is a herbaceous aromatic perennial plant, commonly known as St. John's wort. St. John's wort (SJW) is prepared from the dried aerial parts and flowering tops of *H. perforatum,* and used in both the USA and Europe to manage mild to moderate depression [12, 13, 30]. In the United States, St. John's wort is regulated as a dietary supplement; however, in reality it is used more as a CAM therapy for depression. The major chemical constituents of St. John's wort include the naphthodianthrones hypericin and pseudohypericin and the acylphloroglucinols hyperforin and adhyperforin (Figure 7) [13].

Earlier clinical trials have suggested that standardized extracts of St. John's wort were as effective as low doses of selective serotonin reuptake inhibitors or tricyclic antidepressants for the treatment of mild to moderate depression [13]. Meta-analyses of randomized controlled trials have found that St. John's wort was superior to placebo and similarly effective as standard antidepressants in the acute treatment of mild to moderate depression [31]. In addition, there was a reduced frequency of adverse effects, lower treatment withdrawal rates, low rates of side effects, and good compliance, with St. John's wort as opposed to standard antidepressant drugs [31]. A more recent study demonstrated the cost-effectiveness of using St. John's wort products as a first line of therapy for mild to moderate depression [32]. In general, St. John's wort can interact with many important prescription drugs that are used by patient populations more susceptible to depression such as transplantation patients, HIV/AIDS, women, and cancer patients [33, 34]. The metabolism of drugs such as cyclosporin, protease inhibitors, estrogens, and chemotherapeutic agents is increased when given in combination with therapeutic doses of St. John's wort. Since SJW can interact with any drug metabolized through the cytochrome P450 enzymes, many interactions with prescription medicines are possible, and patients should consult their physician or pharmacist prior to use [31].

## 4. Botanicals in Thailand

Botanicals are important part in the traditional medicine system that was established in China, India, Mesopotamia, and Egypt in 5000 A.C. During 500 A.C.–500 A.D., the traditional medicine systems of Graeco-Roman and Islam evolved and transformed into a modern medicine system in 1800 A.D. [35]. Drugs are defined in modern medicine system as chemicals with well-defined structures, isolated from botanicals or chemically synthesized. For example, vinblastine and vincristine, isolated from *Catharanthus roseus*, are anticancer drugs. Another evolution path of botanicals is the utilization of botanical extracts. Research in botanicals is multidisciplinary as shown by the publications in this current issue. New discoveries from botanicals are going on, while the indigenous and traditional uses of botanicals have been developed and applied in the community. By the beginning of ASEAN Economic Community (AEC) in the year 2015, harmonization of laws and regulations relative to botanicals in ASEAN countries will be established. This encourages each country in the region to improve the laws and regulations relative to botanicals.

Asian countries have a long historical use of traditional medicines and botanicals. They have their own traditional medicinal systems that are accepted by their people and regulated by the states. However, there are some difficulties in integrating botanicals to the national health system. World Health Organization (WHO) has realized the botanical importance in Asia, in which 80% of the populations still rely on traditional medicine system. WHO has developed the guidelines of herbal medicines in the Southeast Asia region for supporting the integration of traditional and modern medicines [15]. In the guidelines, herbal medicines are divided into four categories based on distinguishing the used form and its origin. They are indigenous, traditional, modified, and export/import herbal medicines. Indigenous and traditional herbal medicines use the combination of crude drugs as drug materials. The modified herbal medicine mostly comprises single evidence-based herb in the form of standardized extract, and it is available in the modern dosage form.

The traditional medicine system in Thailand has been developed and influenced by Ayurveda and Chinese traditional medicine systems since the Ayutthaya period (1350–1767 A.D.) [36]. During the reign of King Rama V, Siriraj hospital was established in the year 1888, and the system was a combination of modern and traditional medicine systems. In the year 1913, the traditional medicine system was no longer taught to the medical students owing to the different doctrines and the separation from the modern medicine. The regulation on traditional medicine was issued in the year 1967 under the Drug Act 1964, which is divided into two parts covering modern and traditional medicines.

Because of the return of the botanical importance, the Ministry of Public Health, Thailand, established in 2002 the department involving traditional medicine system. The Department of Thai Medicine and Alternative Medicine, which works together with the Department of Medical Science, issued the list of essential medicines (herbal medicine). The first list was approved in 1999. The latest List, in 2012, covers 52 traditional drugs and 21 single herbal drugs, which are classified as modified herbal medicine and is divided into 4 groups covering the modified Thai traditional medicine, the modified traditional medicine (e.g., ayurveda, Chinese), the single herbal drug, and the modern herbal drug [37]. Currently, there is only one modern herbal drug (silymarin as hepatoprotective) that is licensed because of the strong evidence requirement of safety, efficacy, and quality. It is quite difficult or impossible to prove the efficacy of the botanical products, especially those with mild-moderate effect, with the same standard as the modern medicine. Most of the imported herbal products are licensed as food supplements, of which the medical indication cannot be claimed.

The following are the examples of well-known single herbal products included in the list of essential medicine (Herbal Medicines).

**Figure 8:**
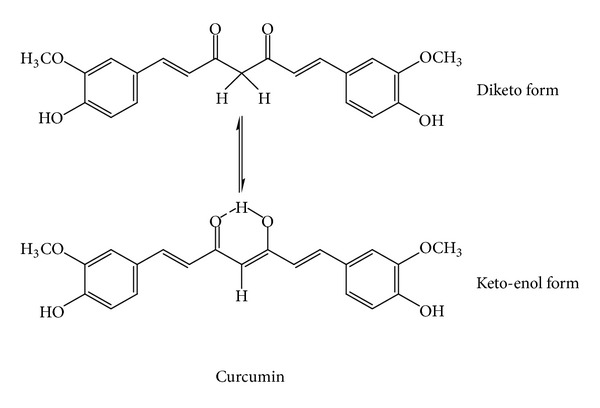
Two forms of curcumin of *Curcuma longa*. The keto-enol form is more preferable [7].

*Andrographis paniculata*, the aerial parts, contained total lactones not less than 6% (calculated as andrographolide, Figure 8). The indications cover the relief of noninfectious diarrhea, with the daily dose ranging from 2 to 8 g and the relief of common cold, with the daily dose ranging from 6 to 12 g. The lactones have weak antimicrobial activity. The therapeutic effect may also be due in part to other activities as well, such as anti-inflammatory and immunostimulatory effects. A review of this botanical is published as a monograph in the WHO Monographs on Selected Medicinal Plants volume 2.

**Figure 9:**
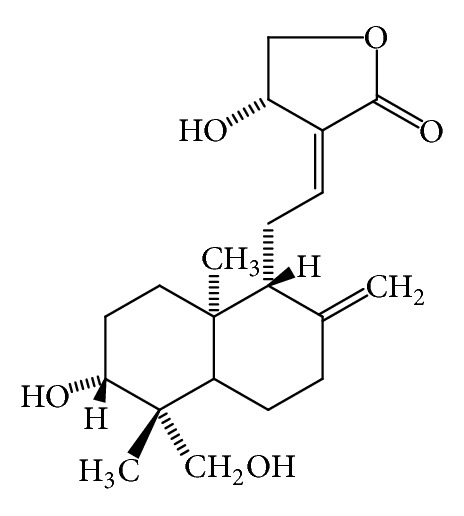
Andrographolide from *Andrographis paniculata* [14].

*Curcuma longa*, the rhizome containing curcuminoids not less than 5% (calculated as curcumin, Figure 9) and volatile oil not less than 6%. It is indicated for the relief of flatulence. The daily dose ranges from 2 to 4 g. The volatile oil is carminative. The curcuminoids (curcumin) in rhizome and cineole in volatile oil stimulate the bile secretion, which aids the digestion and relieves the flatulence. The monograph of *C. longa* was published in the WHO Monographs on Selected Medicinal Plants, volume 1.

Both botanicals are used in the form of powdered crude drug. The used form of drug material determines the method of quality control and the daily dose. The quality control of the crude drug is performed according to the monograph in the Thai herbal pharmacopoeia (THP), which is established by the Department of Medical Sciences, Ministry of Public Health. There are three volumes of THP issued in 1998, 2000, 2007 (supplement), and 2009. They cover 37 monographs of crude drugs [41, 42].

One interesting botanical in the list of essential medicine (herbal medicine) is *Momordica charantia* fruit, which is classified as a single herbal drug and indicated as remedies for fever and aphthous ulcer and as a bitter tonic. The daily dose ranges from 3 to 6 g (infusion) and from 1.5 to 3 g (oral). The use of this botanical is limited to the traditional indication in the list of essential medicine. However, the research on *M. charantia* still continues, and new health benefits from this plant are being revealed.

**Figure 11:**
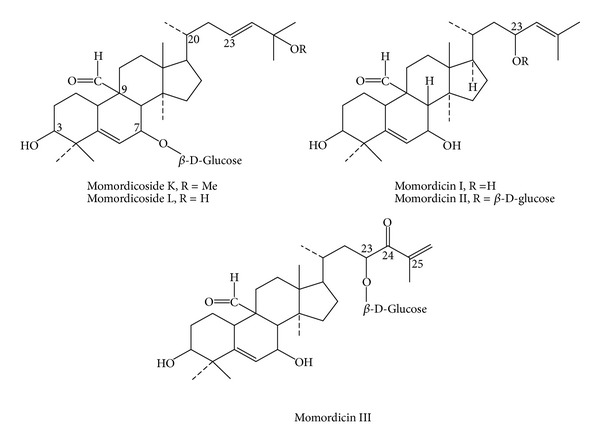
Cucurbitacins from *Momordica charantia* [39].

*Momordica charantia* (bitter gourd, bitter melon) has been used as medicine for thousand years in Africa, Asia, and Latin America. In India, the fruit has been used in ayurvedic medicine as remedies for diabetes, liver disease, gout, and arthritis. In Thai medicine, the leaf has been used as one ingredient in “green recipe,” which relieves fever and the root as remedies for liver disease and blood disorder [43]. Scientific research on *M. charantia* has been continuously performed since 1962, when the antidiabetic substance, charantin, was discovered by Lotlikar and Rao [44]. In 1965 Sucrow identified the structure of charantin as a mixture of sitosteryl- and 5, 25 stigmastadiene-3*β*-ol-D-glucoside (in the ratio 1 : 1, Figure 10). Baldwa et al. isolated the insulin-like compound from the fruit in the year 1977 [45]. Khanna et al. identified the insulin-like compound as a polypeptide with the molecular weight of 11 kD, 166 amino acids, and named it polypeptide-P [39]. The plant also contained cucurbitacins (Figure 11), and they were momordicosides, momordicines, karavilosides, and charantosides [46]. These cucurbitacins have antidiabetic effects [47]. *M. charantia* fruit contained several antidiabetic compounds and lowered the blood sugar via several mechanisms such as the stimulation of insulin secretion from pancreas, the decrease of sugar formation from the liver, the increase of glycolysis, the renewal of pancreatic *β* cells, the increase of insulin sensitivity, the increase of glucose tolerance, and the inhibition of *α*-glucosidase [48–52]. *M. charantia *affects carbohydrate and lipid metabolism through the stimulation of thyroxine and AMPK (AMP-activated protein kinase) [53]. It can also suppress the insulin-signaling pathway resulting in the increase of insulin sensitivity [54].

The *in vivo* studies of the fruit juice of *M. charantia* were given to rabbits and mice, showing antidiabetic effect [55–58]. Some reports stated that *M. charantia* could alleviate the complications resulting from the long time uncontrolled blood sugar level, such as kidney damage, cataract, and peripheral neuritis [59–61].

One clinical study in type II diabetic patients showed that *M. charantia* fruit improved glucose tolerance, decreased the postprandial hyperglycemia, and lowered the urination frequency [62]. The diabetic patients normally suffer from hypertriglyceridemia, hypertension, and obesity, and thus, *M. charantia *fruit seems to be an appropriate complementary and alternative medicine because of the presence of bioactive compounds, which are responsible for the lowering of blood sugar, blood lipids, blood pressure, and body weight [52, 63–66]. Currently, the clinical evidence of *M. charantia* fruit as an antidiabetic agent is insufficient. Such clinical results usually occur with the food plants that have mild-moderate activity. Well-designed clinical trials and quality control as well as production process control are recommended for further clinical investigation. The increase of prediabetes and diabetes incidences caused a great burden on the healthcare expense. The use of nonmodified botanicals for self-medication like the fruit juice of *M. charantia* may be a good choice of complementary and alternative medicines, and it is cost-effective [40, 67].

**Figure 12:**
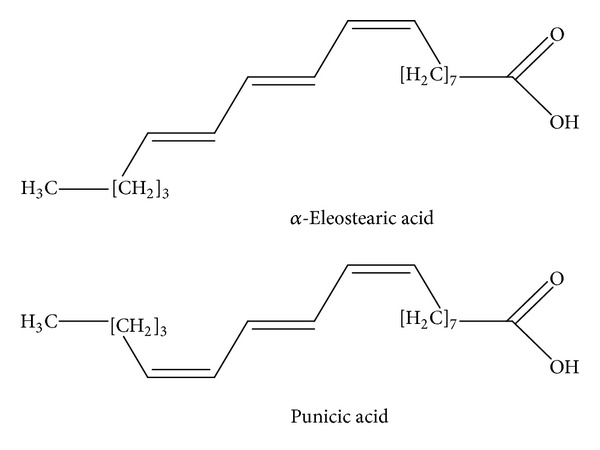
*α*-Eleostearic acid and punicic acid from *Momordica charantia* [40].

Other parts of *M. charantia *have also been investigated for the medicinal purposes. The seed oil contained conjugated linolenic acid, namely, *α*-eleostearic and punicic acids (Figure 12), which are strong antioxidant and anti-inflammatory agents [68].

The seed oil could decrease the blood lipids and prevent cardiovascular disease [69]. The seed proteins, *α*-momorcharin, Momordica anti-HIV protein 30 (MAP30), and Mara Khee Nok 29 (MRK29), which are type I ribosome inactivating proteins (RIPs I), have antitumor, antiviral, and anti-HIV effects [70–72]. According to the strong cytotoxicity of cucurbitacins, *M. charantia* has been also intensively investigated for its anticancer effect. It could inhibit several cancer cell lines, for example, adrenocortical, breast, nasopharynx, and prostate cancer cell lines [40, 73–76].

## 5. Summary

Botanicals have been recognized as food and medicines in every country in the world for thousands of years. The progress of science and technology and the vast amount of research on botanicals have given rise to the evolution of botanical utilization. Herbal products are regulated under different laws on different continents, and harmonization of some of these laws would be beneficial to everyone. Botanical extracts have been developed and are used as active composition in HMPs (traditional and modern) including food supplements in EU and dietary supplements in USA, which are classified as not drugs or food. In Thailand, the use of a single or combined crude drugs as traditional medicine still exists. However, modern botanical products (HMPs/phytopharmaceuticals, food supplements, and dietary supplements), in which the quality, effectiveness, and safety are supported by clinical data, and the understanding of the modern practitioners should be integrated into the modern drug system. This would make for a better healthcare system globally and make it cost effective. In addition, research on botanicals will eventually give rise to the discovery of new drugs, providing that there is sufficient funding for such research.

## Figures and Tables

**Figure 10:**
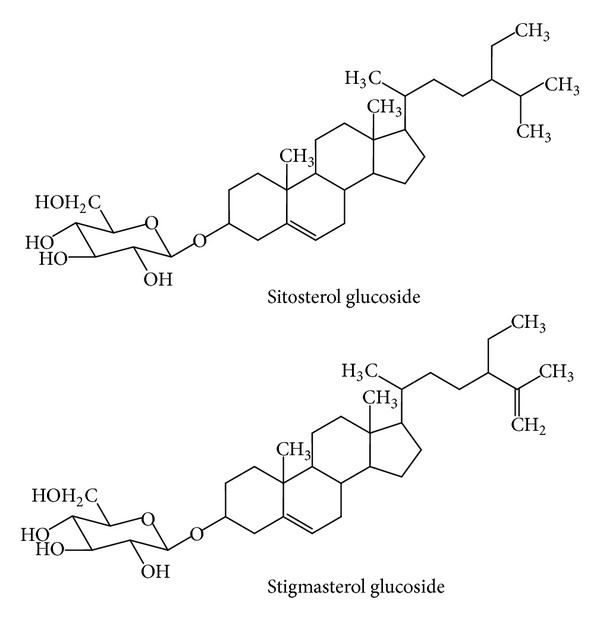
Charantin (a mixture of sitosterol and stigmasterol glucosides, 1 : 1) from *Momordica charantia* [38].
